# The experience of gestational diabetes for indigenous Māori women living in rural New Zealand: qualitative research informing the development of decolonising interventions

**DOI:** 10.1186/s12884-018-2103-8

**Published:** 2018-12-05

**Authors:** Jennifer Reid, Anneka Anderson, Donna Cormack, Papaarangi Reid, Matire Harwood

**Affiliations:** 1 c/- Te Kupenga Hauora Māori, Medical and Health Sciences, University of Auckland, 261 Morrin Rd, Glen Innes, Auckland, 1072 New Zealand; 2 Te Rōpū Rangahau Hauora A Eru Pōmare, University of Otago, 23A Mein St, Newtown, Wellington, 6021 New Zealand; 3 National Hauora Coalition, Units 3-4, 485B Rosebank Rd, Avondale, Auckland, 1026 New Zealand

**Keywords:** Gestational diabetes, Type 2 diabetes, Indigenous, Māori, Decolonising, Kaupapa Māori, Mana Tū

## Abstract

**Background:**

Although early detection and management of excess rates of gestational diabetes mellitus (GDM) among Indigenous women can substantially reduce maternal and offspring complications, current interventions seem ineffective for Indigenous women. While undertaking a qualitative study in a rural community in Northland, New Zealand about the complexities of living with diabetes, we observed a common emotional discourse about the burden of diabetic pregnancies. Given the significance of GDM and our commitment to give voice to Indigenous Māori women in ways that could potentially inform solutions, we aimed to explore the phenomenon of GDM among Māori women in a rural context marked by high area-deprivation.

**Method:**

A qualitative and Kaupapa Māori methodology was utilised. A sub-sample of women (*n* = 10) from a broader study designed to improve type 2 diabetes mellitus (T2DM) who had experienced GDM or pre-existing diabetes during pregnancy and/or had been exposed to diabetes in utero were interviewed. Participants in the broader study were recruited via the local primary care clinic. Experiences of GDM, in relation to their current T2DM, was sought. Narrative data was analysed for themes.

**Results:**

Intergenerational experiences informed perceptions that GDM was an inevitable heritable illness that “just runs in the family.” The cumulative effects of deprivation and living with GDM compounded the complexities of participant’ lives including perceptions of powerlessness and mental health deterioration. Missed opportunities for health services to detect and manage diabetes had ongoing health consequences for the women and their offspring. Positive relationships with healthcare providers facilitated management of GDM and helped women engage with self-management.

**Conclusion:**

Māori women living with T2DM were clear that health providers had failed to intervene in ways that would have potentially slowed or prevented progression of GDM to T2DM. Participants revealed missed opportunities for appropriate diagnostic testing, treatment and health promotion programmes for GDM. Poor collaboration between health services and social services meant psychosocial issues were rarely addressed and the cycle of intergenerational poverty and disadvantage prevailed. These data highlight opportunities for extended case management to include whānau (family) engagement, input from social services, and evidence-based medicine and/or long-term management and prevention of T2DM.

**Electronic supplementary material:**

The online version of this article (10.1186/s12884-018-2103-8) contains supplementary material, which is available to authorized users.

## Background

Indigenous women are at greater risk for developing gestational diabetes mellitus (GDM) than non-Indigenous women. Rates among Native American (7.9%), Canadian First Nations (11.5%), Indigenous Australians (8.4%), and Māori in Aotearoa/New Zealand (New Zealand) (6%) are higher than those of non-Indigenous women in those respective nations and the total international incidence (2–5%) [1–3].

The short-term effects of GDM are well documented [4] and our understanding about the long term consequences for mother and child is growing as results from longitudinal studies become available [5, 6]. For mothers, GDM is associated with perinatal complications, maternal obesity, T2DM [7–9] and cardiovascular disease (CVD) [10, 11]. GDM is also associated with an increased life time risk for offspring becoming obese or developing T2DM [12]. Seminal studies among Pima Indians, a Native American population with the highest international incidence of GDM [13], and one of the highest incidence T2DM in adults and children [14] and obesity [15], found diabetic pregnancies were ‘responsible’ for 40% of T2DM in offspring (5–19-years) [16].

For GDM, the argument that “diabetes begets diabetes” and other long term conditions [17] is compelling for Indigenous people [18]. In New Zealand adults, Māori self-reported rates of T2DM are double that of non-Māori [19]; and the incidence of T2DM has increased five-fold in children under 15-years but is significantly greater for Māori than NZ Europeans (3.4 cf. 0.1 per 100,000) [20]. Substantial disparities also exist in rates of obesity between Māori and New Zealand European adults (46.5% cf. 25.7%) and children (5–14 years) (15.7% cf. 6.7%) respectively. Māori childhood overweight and obesity after exposure to GDM in utero is strongly associated with subsequent development of T2DM and CVD [2]. Further, GDM is a significant predictor of maternal CVD [10], the leading cause of mortality among Māori females [21].

Critically, early detection [22–24] and intervention of GDM can substantially reduce maternal and offspring complications [13]. Yet GDM is considered a complex condition [10] and difficulties exist in quantifying causal factors which affects the development and implementation of screening, diagnosis and management protocols [4, 13] particularly for marginalised peoples [25, 26]. While GDM susceptibility is attributed to genetic, social and environment factors [13], genetic variation in T2DM risk probably only very modestly contributes to the predicted risk of GDM [27]. Despite extensive research over the past twenty-years identifying T2DM as a polygenetic disease, the potential epigenetic interactions between genes and the environment in Indigenous populations remains poorly understood [28, 29].

Most experts agree that a focus on “ethnicity as a proxy for genetic variation” being a primary determinant of GDM risk diverts attention away from the wider determinants affecting Indigenous peoples’ health [30]. In fact, the contribution of socioeconomic and psychosocial deprivation to GDM prevalence among Indigenous women has not been adequately evaluated [31]. The experience of “unequal treatment” [29] for Indigenous women with GDM also receives little attention, despite several small studies and a recent audit reporting inequities in screening, diagnosis and management of GDM between Māori and non-Māori [22, 32, 33]. This research highlights the need for qualitative data that not only provides rich and meaningful information, but can also be a powerful tool for change [34] and to inform the development and implementation of effective interventions [35].

The paucity of qualitative research exploring Indigenous women’s experiences and perceptions of GDM [11, 36–38] was highlighted for us when conducting research on T2DM. While undertaking a qualitative study in a rural Māori community about the complexities of living with diabetes, we noted that a common, often emotional, discourse about the experience of diabetic pregnancies was emerging from the data.

Our participants lived in Northland, New Zealand, a site chosen for its unique sociodemographic and geographical issues. There is high regional deprivation in Northland that is experienced more by Māori than non-Māori (39% cf. 14%) [39]. Māori in Northland are markedly disadvantaged compared to non-Māori across all socioeconomic indicators, including educational attainment, unemployment status and income level and are more likely to live in rented accommodation and overcrowded households [39]. Geographic barriers to accessing health care are also notable in Northland [40]. Access to secondary diabetes care services was 150 km away from the community we were working with.

Although Māori represent only 26% of Northland’s female population of childbearing age, McGrath and Baldwin found that 56% of those diagnosed with GDM and available for follow-up at 2-years had developed T2DM or impaired glucose tolerance (IGT); over 33% of these Māori women, aged in their thirties, probably had undiagnosed pre-pregnancy IGT [33]. Given the significance of the health issue and a commitment to give voice to Indigenous Māori women in ways that could potentially inform solutions, we aimed to explore the phenomenon of GDM for Māori women living in a rural community.

## Methods

The primary aim of this study was to investigate the experience of GDM amongst a sub-sample Māori women, enrolled in a larger pilot study designed to improve T2DM outcomes. The secondary aim was to develop potential solutions to the challenges identified by participants. This research adopted qualitative interview methods to explore the perceptions, attitudes and emotions [41] of Māori women with T2DM around their experience with GDM. The researchers utilised a Kaupapa Māori research methodology [42–45]. Kaupapa Māori research seeks to benefit Māori through critiquing socio-political systems that create inequities for Māori and by rejecting victim blaming and deficit theories [46]. This framework prioritises Māori ways of knowing and Māori realities [45, 47].

The data drawn on for this paper derived from ten participants who were a sub-sample of a larger study (*n* = 27) undertaken with Māori living with T2DM who resided in the selected community within Northland. The main inclusion criteria for the larger study were: aged 18-years and older; having a glycated haemoglobin (HBA1c) > 53 mmol/mol; and being enrolled with the local primary care clinic. The ten participants advised that they either had a clinical history of GDM (*n* = 8) or had been exposed to diabetes in utero (*n* = 2). Eligible participants were recruited via the local primary care clinic between September and December of 2015.

For the larger study, a Māori nurse at the local primary care clinic contacted eligible participants, providing them with information about the research and invited them to participate. The contact details of all consenting participants were then provided to the lead researcher, who contacted participants via phone or home visits (when no phone service was available). The lead investigator provided detailed information about the research, answered any questions from women and obtained written informed consent and confidentiality agreements (including accessing their diabetes-related health data from the clinic) for each participant. Thirty-three participants initially agreed to take part in the larger study and six have withdrawn over the duration of the study, primarily because of moving from the area.

Open ended, semi-structured, face-to-face interviews were undertaken with the ten women due to their ability to capture rich narrative data [48] (Interview Schedule refer Additional file 1). Interview questions were based around participants’ experiences of T2DM, and GDM. Interviews were audio recorded and lasted approximately forty-minutes. They were undertaken in participants’ homes or the health centre, or a mutually agreed venue. The lead researcher transcribed the audio recorded interviews and participants were invited to review, edit or change their transcript prior to data analysis.

A general inductive approach was used to analyse the data from repeated readings of the transcripts, to identify and produce categories and themes that aligned with the research objectives [49]. The lead researcher independently coded the data [50], and discussed the codes and themes with two experiences Kaupapa Māori researchers, who had access to the interview data.

As this study involved human subjects, ethnical approval could not be provided by the University of Auckland but was received from the NZ Health and Disabilities Ethics Committee HDEC Northern A (15/NTA/107) on 19/08/2015. The primary care provider involved in this study signed a memorandum of agreement with the University of Auckland on 27/07/15. The Health Research Council of NZ funded this study as part of a post-doctoral award (15/428); funding covered the design of the study, and the collection, analysis and interpretation of data, and writing of the manuscript by the primary author. Consent for data used in this study was cover by consents for the larger study. Mana Tū Trial Registration No: ACTRN12617001276347

## Results

The data was from drawn from ten Māori women aged between 30 and 69 years. As outlined in Table 1, participants had diverse pregnancy experiences and current diabetes-related health conditions. Data were de-identified after collection and participants were allocated codes for analysis and pseudonyms for publication. Most participants also reported a family history of diabetes, including having children with diagnosed GDM and/or T2DM in puberty or young adulthood.

**Table 1:** GDM and TD2M across the generations

No	Name	Age Range	Family history of diabetes	Diabetes Pre-pregnancy	Diabetic pregnancies	Parity	Diagnosed Diabetes in offspring to date
1	Huhana	50–59	Mat	No	1st GDM	4	2 T2DM
					2nd T2DM		2 pre-T2DM
3	Rahera	60–69	None	No	3rd GDM	6	None
					6th T2DM		
9	Erana	50–59	Mat	16-yrs	all	5	1 T2DM 14-yrs
11	Anahera	40–49	Mat	No	Don’t know	5	2 GDM
13	Aiwi	30–39	None	No	2nd GDM	4	4 children none
					4rd T2DM		
15	Hutita	50–59	Mat Pat	No	2nd GDM	3	none
					3rd T2DM		
19	Ema	60–69	Mat	No	No GDM	6	2 GDM
21	Aira	60–69	Mat	18-yrs	Unsure	7	3 T2DM
26	Rea	60–69	Mat	No	No DM	3	1 T2DM
28	Arihi	40–49	Mat Pat	No	5th GDM	8	1 adult no
					8th T2DM		7 children no

Participants’ understandings and experiences of GDM were influenced by their family histories of GDM and from their exposure to deprivation and complex living contexts. The women’s narratives revealed that the diagnosis and management of their GDM were hindered through missed opportunities in the health system and through poor communication and rapport with health care professionals and health care services. Despite these barriers, it was evident that participants went to great lengths to try and manage their GDM.

### Family histories of T2DM, perceptions and understandings of GDM

Participants expressed varied understandings and perceptions of GDM. Many participants understood T2DM to be an inherited illness and one participant attributed birth parity as a causal factor. Understandings and perceptions were influenced by long histories of exposure to family members with T2DM and/or GDM.

As evident in Table 1, many (*n* = 8) participants had family histories of T2DM. Many participants themselves were exposed to diabetes and obesity in utero and had children with early onset T2DM. For example, Huhana’s eldest son, Hayden, was exposed to gestational hyperglycaemia and was born with neonatal hypoglycaemia. Hayden was diagnosed with T2DM at the age of 23 after he collapsed at work. Likewise, Erana was diagnosed with adolescent-onset T2DM. Erana’s daughter, Ella, was born pre-term and low birth weight, and at 14-years of age, Ella was also diagnosed with early-onset T2DM. As Erana described, “*Ella’s got diabetes real bad…She kept collapsing [at school and was hospitalised]. She was meant to go on a camp for diabetic kids…but she was too sick*”.

These intergenerational experiences informed perceptions that GDM was an inevitable, heritable illness that “*just runs in the family*”. For example, Anahera stated that she knew she would get GDM because five generations of women in her whānau had each experienced GDM:

My mother had diabetes. I knew I was going to get it because…my [maternal] grandmother and her mother had it before her, so it just runs in the family…when diagnosed I didn’t really think much of it… [two out of three of her daughters] they were both thinged [affected] with [GDM], well whatever… they don’t have it fully yet.

The understanding that GDM is a heritable disease was reinforced for some women by racialised discourse from their health care providers who described it as a ‘Māori condition’, as Aiwi explained:

When I was first told [she had T2DM after the birth of her fourth baby], it was like: “Oh well, you’re Māori, it was bound to happen”. And I thought but I haven’t got it on my mother’s or father’s [side of the family].

Witnessing the many adverse effects that T2DM and/or GDM had on family members’ lives, including their relationships with whānau, and physical, emotional and behavioural impacts created perceptions of fear of GDM for participants. Huhana explained that seeing the impacts of T2DM on both her grandmother and mother made her fearful as she believed it would prevent her from having a happy life:

My [maternal] grandmother was a diabetic…. [My mother] was the last of four sisters… [to die from] complications with their diabetes…. They were the most [angry] women and they took it out of everybody around them and I realise now it was because they had low blood sugar or they were hypo…. I didn’t want to be like that…. I just wanted to live life, be happy not have to worry about it…. [The specialist] told me: “you’ve got GDM…” it was scary because it was my first pregnancy and I didn’t know about diabetes, I mean, I knew, but I… wasn’t informed.

### Contexts of deprivation

It was evident from participants’ narratives that structural determinants of health created complex living situations for them that compounded the difficulties they experienced with GDM. The majority of the women were exposed to contexts of deprivation, particularly poor housing conditions, household crowding and poverty. The costs and geographic distances associated with accessing health services proposed huge challenges for participants and created barrier to access, as Huhana’s narrative illustrates:

I got pregnant one to two-years later and this time I was huge. The specialist scared me because there’s this little white woman saying to me, and I’m a big black Māori woman “if you don’t control this… either you or your baby are going to die”. Everybody [clamped] down, it was appointments [at hospital which was a 90-minute drive]. I wouldn’t make them I just couldn’t afford it, we had no vehicle that was legal.

Aiwi also reported being stressed by the need to prepay costs associated with travelling to specialist diabetes care:

I’ve got to scrape up $40 or $50 just to get down there and back for a 10-minute appointment at the hospital and then it was 10 to 14-days before you got that reimbursement back.

The cumulative effects of deprivation and living with GDM compounded the complexity of participants’ lives and their narratives show how this stress manifested for them in many ways, including feeling that they had no control in their lives, creating strain on relationships with their partners and other whānau members, and the onset of depression as Huhana described:

With the fourth pregnancy, I had sort of lost control. I was living in the garage with my three children [and husband]. I was huge and six-months [along in my pregnancy] when I was diagnosed with depression…. I didn’t want to do anything. I’d just wake up [and] went through the motions.

### Health system barriers to the detection, treatment and management of GDM

It was evident from participants’ narratives that despite their risks of developing GDM, there had been a lack of screening and vigilance for the detection of diabetes during their encounters with health services and health care professionals. For example, Anahera’s two elder daughters’ both experienced GDM with all their respective pregnancies. Despite her second daughter, Bev, having a history of GDM with both pregnancies, a nurse refused to give her a postpartum diabetes test because she was under the age of 35-years:

If you want to get checked for diabetes, they [health care professionals] should do it, doesn’t matter whether you’re not 35… [Bev] always gets [testing] done when she comes up [to the health centre] … just coz she wants to make sure that she doesn’t have diabetes.

In addition, when Anahera’s third daughter, Cathy, became pregnant, Anahera herself was vigilant around GDM screening for her daughter and when this was not undertaken by her midwife, she advocated Cathy seek another midwife, as Anahera explained:

[Cathy] she’s only seen [the midwife] maybe twice so hasn’t been checked [for GDM] so we’re going to try and get her another midwife…She would have been checked around October and then maybe a couple of weeks after but she’s supposed to be checked, well, nearly every week now because she’s due [in mid-January].

These missed opportunities for health services to detect diabetes had on-going consequences for the health of the women and their babies. For example, Aiwi described the impact of potentially undiagnosed GDM on her first premature birth and second pregnancy with GDM;

The first baby was … premature. I think it’s around the 26-week mark [when] they test for diabetes in pregnancy? And because I hadn’t reached that far yet I missed the testing. They never found anything in his placenta…. It wasn’t until I got diagnosed with GDM in the second pregnancy that they sort of assumed, “Okay, this might be why you had your prem. baby”.

If GDM was diagnosed in Aiwi’s first pregnancy, it could have helped to better mitigate the premature pregnancy and potentially aid the diagnosis and management of GDM for her second pregnancy. Another area of health care where there was important missed opportunity to improve GDM outcomes for women was the notable lack of attention to addressing risk factors of GDM. Many of the participants described experiencing risk factors, including pregravid obesity, excess gestational weight gain, and postpartum weight retention that had not been adequately addressed by health care professionals or adequately considered in the management of their care. For example, Aiwi disclosed that “in my third pregnancy my weight skyrocketed to 157 kilos, but then she [her baby] was 4,808g”.

Likewise, Arihi also described her incremental weight gain across her eight pregnancies: “I was a size 12 about 20-years ago. I was able to wear his [her partner’s] jeans when I was pregnant and get back in my own when I was dropped. I can’t do that anymore”. The experiences of pre-existing diabetes described by Erana below highlight the impact of not addressing key risk factors, such as excessive gestational weight gain, that potentially lead to avoidable adverse health outcomes:

With my first child, I was slim…. then I was huge. I couldn’t hold my guts because I was too heavy. I just couldn’t feel my feet; they were just numb and swollen. My hands were swollen I had to get my ring cut-off my finger.

In addition to these gaps in health services around GDM, poor communication of health information from health care providers created confusion and barriers to health care for many participants. Participants were often not clearly informed why they were having tests or what different tests result meant for their health. Arihi’s narrative below illustrates her confusion over GDM testing for her fifth pregnancy which made her feel as though she was “not in charge” of her own health:

[My fifth pregnancy] was when they told me I had GDM. The first and second were fine. The third one, I remember doing that sugar drink [GDM test] but my results came back fine. With the fourth one, I done the sugar drink twice because the first time it came back showing something, so I did it again just to make sure, and it came back fine. So [I was] a bit confused.

### Facilitators of GDM management

In contrast to the barriers to effective diabetes screening and management identified in the research, the willingness of participants to manage their GDM and associated risks, including undertaking tests, was a key facilitator of their experiences. Huhana described how she overcame her fear of injections to facilitate the insulin management of her diabetes:

It was scary for someone to tell you to poke your stomach when you had a baby in there. [With the first pregnancy] I refused to. They [health care professionals] got me to inject oranges, what the hell’s that going to do? I couldn’t even take my finger prick, that’s how petrified I was of needles. [After the second birth] I had to start to inject myself just during the day. With the third baby, I was injecting into my thigh. I used to gear myself up, just breath for about 10 minutes and go “Okay I can do this” and [I] learnt through trial and error.

Having access to resources such as diabetes workshops also facilitated the management of GDM for participants as explained by Rahera:

Well, I drank coca cola, like [eight litres daily] …they used to have classes [at the health centre] for diabetics and when they showed us exactly what went into coca cola it kind of made me think I need to get off this - so I worked hard towards that. We had a good diabetes nurse too back then and she used to even visit the homes just to make sure that we were going on the right track… she helped me a lot.

Arihi’s quote below demonstrates how having good relationships with health care providers facilitated her transition from specialist care to primary health care services and diabetes management:

I used to get phone calls all the time from [staff at the diabetes centre] even afterwards, they’d follow up until I got back to [the health centre]. They were calling me during my pregnancy and in between my appointments, and afterwards too, making sure I’ve got everything and I’ve made my arrangements.

The importance of good communication and rapport between the diabetes specialist and women was also evident in Huhana’s narrative:

[The specialist] made clinics [closer to her home] …. She just hammered it into me “you need to do this, you need to do that, you need to change that.”

## Discussion

In describing their experiences of GDM, Māori women living with T2DM in Northland were clear that health providers had failed to intervene in ways that would have potentially slowed or prevented progression of GDM to T2DM [13]. Participants revealed missed opportunities for appropriate diagnostic testing, treatment and health promotion programmes for GDM. Furthermore, engagement by health services with family and social services was poor. As a result, psychosocial issues were rarely addressed and cycles of intergenerational poverty and disadvantage prevailed.

Such breaches of Indigenous women’s rights to health and social services [51] underscore United Nation’s recognition of colonisation as the most important social determinant of Indigenous health [52, 53]. Upstream colonial processes (political, social and economic), both historical and contemporary, are causally associated with the deprived contexts that initiate and sustain the disproportional burden of GDM experienced by Indigenous women [29]. Study findings have provided important information to inform development of interventions. We see opportunities for evidence informed case management, whānau engagement, input from social services, evidence-based medicine for long-term management of T2DM, including access to bariatric surgery when appropriate. Equally, findings reinforce that interventions must occur within a decolonising framework [45].

Case management for poorly controlled T2DM is effective with individual improvements in HbA1c and low-density lipoprotein (LDL) levels [54]. Others have described the potential benefits of case management for GDM to service pathways, including improved transfer from care in the community to diabetes specialist and hospital-based services, and the seamless and timely resumption of local general practitioner (GP)-led postnatal care, and appropriate referrals to social services that address broader social issues (e.g. housing) [55]. However, the findings from our research suggest extending case management beyond the individual to include the family.

Oster et al. [11] recommend family-based case management programmes for the care of First Nation’s women’s pregnancies, arguing that such care responds to individual identities, experiences and perspectives, while simultaneously utilising the whānau to disseminate knowledge, engender support, and enhance awareness of risk factors. The fact that opportunities to address GDM in our cohort were missed is unacceptable, particularly as pregnancy is considered an important “teachable moment” [56] for mothers who are highly motivated to safeguard the health of their unborn child [57]. Sound family engagement is critical to such ‘opportunities,’ particularly when it facilitates broad dissemination of diabetes-related knowledge and recruits spousal, whānau and community support [58]. The wider benefits of whānau engagement, including the reduced likelihood of postpartum depression [59] and other common stress-related conditions [60], are well established.

Findings also highlighted the failure to provide systematic, targeted screening and preconception education and counselling of young Māori women and their whānau [57] in addition to culturally safe care and follow-up [24, 61]. The literature and our findings suggest that robust referral pathways to social services are essential [62]. Consistent with best-practice recommendations from the International Association of Diabetes and Pregnancy Study group [63] testing criteria must be regularly evaluated to ensure that it achieves quality results, including equity in GDM short and long term outcomes for Indigenous people [10, 64].

Finally, interventions must sit within a decolonising framework that empowers women, rejects deficit victim blaming discourses and recognises contexts of deprivation. Plans to test a novel decolonised intervention – Mana Tū - within the context of primary care are underway in this rural community. Mana Tū was developed by a Māori-led collaborative of primary health care workers and researchers, and co-designed with whānau (patients and their families) to support Māori and other people living within contexts of deprivation with poorly controlled diabetes (HbA1c > 65 mmol/mol) assume greater control of their diabetes. Crucially, Kai Manaaki (skilled case managers) employed to work with whānau with diabetes and prediabetes can direct whānau to central hub for referral to a cross-sectorial network of services to address the broader determinant of health.

### Strengths

To our knowledge, this is the first qualitative study to explore GDM and diabetes in pregnancy in New Zealand. Importantly, it describes the outcome of exposure to diabetic pregnancies across several generations. Further, the use of purposive sampling of a small number of diverse, information rich cases provides insights and in-depth understandings of Indigenous women’s experiences [65] to inform decolonising interventions.

### Limitations

This study has several limitations. First, these findings should be viewed in the context of a small sample (*n* = 10) from one high deprivation, rural area of New Zealand and may not be generalizable to other Māori women. Second, self-reported data are inherently subjective [66, 67] and may be affected by memory decline or recall bias, the desire to provide socially acceptable responses and the personal/group discrepancy [68].

## Conclusion

Our study highlights the challenges of preventing, halting or at least slowing the progression of GDM to T2DM in Māori women and suggests extending case management beyond the individual to include the family. The implementation of Mana Tū in a rural primary care clinic in Northland is delivering Māori-centred case-management of diabetes and providing access to cross-sector services to address the wider social determinants of Indigenous health equities. The projected demographic drivers of Māori diabetes - lower average age of diabetes onset and increasing numbers of young Māori women of reproductive age, exposed to diabetes and/or obesity in utero [39] - reinforces the urgency of such Māori-led initiatives.

## Additional file

Additional file 1: Interview Schedule for participants. Guide to questions. (DOCX 25 kb)

## Abbreviations

CVD: cardiovascular disease
GDM: Gestational diabetes
HbA1c: glycated haemoglobin
Mat: maternal
Pat: Paternal
T2DM: type 2 diabetes mellitus
