# Supplementary material for: The experience of gestational diabetes for indigenous Māori women living in rural New Zealand: qualitative research informing the development of decolonising interventions
Source: BMC Pregnancy Childbirth. 2018 Dec 5;18:478. doi: 10.1186/s12884-018-2103-8 (PMC6282285; doi:10.1186/s12884-018-2103-8)
Supplement: Supplementary file 1 — Interview Schedule for participants. Guide to questions. (DOCX 25 kb) [file 12884_2018_2103_MOESM1_ESM.docx]

# INDIVIDUAL PARTICIPANT INTERVIEW SCHEDULE

This schedule represents a loose guide for questions that will asked during face-to-face interviews with study participants. The overall aim is to explore the complexities that impact on the lives of participants by investigating the impact of structural-, provider, and person-level barriers to optimal diabetes management, and the impact of racism(s) on both intergenerational and life course access to the determinants of health. While some questions are informed by the literature, others will be individualised to probe participants’ responses to questionnaires and survey instruments.

## Introduction & context

Can you tell me a little bit about yourself:

- Time spent living in Whangaroa?
- Occupation?
- Source of income?
- Leisure activities?

## Diabetes history

When was your diabetes diagnosed (where, by whom, how, treatment insulin/pills)?

How did you feel and what did you think about diabetes at the time?

How much did you know & what did you believe about diabetes then?

What did you whānau (extended family) feel and think about your diagnosis?

How did diabetes impact on your social life and you as a person?

How would you describe your feelings and thoughts about diabetes now?

What have been the main changes in your experience of diabetes? (e.g. Insulin)

Have you ever been hospitalised because of your diabetes? (when, where, why)

Did any of your tipuna (ancestors), or do any of your whānau (extended family), have diabetes?

## Management & self-care

Individualise probe with responses to:

1. Diabetes Attitudes Survey (DAS)
2. Problem Areas in Diabetes (PAID)
3. Diabetes Empowerment Survey (DES – Short Form)
4. Brief Diabetes Knowledge Test (BDKT)
5. Kessler 10 test (K-10)
6. Kupenga Māori Social Survey 2013 and NZ Health Surveys

- Management of diabetes?
- Dietary modification?
- Regular physical activity?
- Self-monitoring blood sugars (BS)? (frequency, who, does it change meal timing/content)
- Medication (use, beliefs, side-effects, types & changes to dosage, costs)
- Social support - whānau helps with your self-care?
- Do you check your blood sugar? (how often)
- Do you check your feet? (how often, podiatrist check)
- Do you have your eyes checked?
- What is the hardest thing about self-care?
- Who helps you the most in caring for your diabetes?

## Intergenerational & life course assess to the determinants of health

The following factors will be used to explore the potential relationship between lifecourse exposure to racism and health (Gee, Walsemann, & Brondolo, 2012)

- Age-patterned exposures: pathways of individuals throughout lifecourse impact of historical events (e.g. urbanisation) and structuring of social institutions (e.g. education). Probe: exiting and entering different social institutions during ageing process.
- Sensitive periods: probe specific development stages (e.g. early childhood, maternal health).
- Linked lives: interdependence of whanau, probe whether exposure to discrimination weakened and/or strengthened social relationships.
- Latency periods: (e.g. long latency period associated with some health outcomes and discrimination).
- Stress proliferation: single stressor can cause proliferation of secondary stressors (e.g. unemployment = financial strain = martial strain).
- Historical period & birth cohort effect (e.g. urban migration, neoliberalism).

## Health provisions

- How would you improve your health services?
- Accessibility of nurse – doctor?
- Communication with nurse – doctor?
- Relationships with nurse – doctor?
- Improved HP transfer of knowledge?

## Barriers

What kind of supports or resources from the government would help you better manage your diabetes?

- System-level (income support, housing).

What kinds of community or local support and resources would help you better manage your diabetes?

How much extra money or what would it take to make your diabetes a priority?

What kind of things would help you to self-manage your diabetes?

- Learning to deal with depression and distress,
- Learning more about self-managing diabetes,
- Learning to relax and de-stress,
- Learning other ways of thinking about diabetes.

If you could change one thing about living with diabetes, what would it be?

---------------------------------------------------

Questions outlined in this schedule have been influenced by the literature (Graffigna, Barello, Libreri, & Bosio, 2014; Protudjer, Dumontet, & McGavock, 2014; Ritholz, Beverly, Brooks, Abrahamson, & Weinger, 2014).

## References

Gee, G. C., Walsemann, K. M., & Brondolo, E. (2012). A life course perspective on how racism may be related to health inequities. *Am J Pub Health, 102*(5), 967-974.

Graffigna, G., Barello, S., Libreri, C., & Bosio, C. A. (2014). How to engage type-2 diabetic patients in their own health management: implications for clinical practice. *BMC Public Health, 14*(648).

Protudjer, J. L., Dumontet, J., & McGavock, J. M. (2014). My voice: a grounded theory analysis of the lived experience of type 2 diabetes in adolescence. *Can J Diabetes, 38*(4), 229-236.

Ritholz, M. D., Beverly, E. A., Brooks, K. M., Abrahamson, M. J., & Weinger, K. (2014). Barriers and facilitators to self-care communication during medical appointments in the United States for adults with type 2 diabetes. *Chronic Illness, 10*(4), 303-313. doi: 10.1177/1742395314525647
